# Generation of Induced Pluripotent Stem Cells from Human Nasal Epithelial Cells Using a Sendai Virus Vector

**DOI:** 10.1371/journal.pone.0042855

**Published:** 2012-08-13

**Authors:** Mizuho Ono, Yuko Hamada, Yasue Horiuchi, Mami Matsuo-Takasaki, Yoshimasa Imoto, Kaishi Satomi, Tadao Arinami, Mamoru Hasegawa, Tsuyoshi Fujioka, Yukio Nakamura, Emiko Noguchi

**Affiliations:** 1 Department of Medical Genetics, Faculty of Medicine, University of Tsukuba, Tsukuba, Ibaraki, Japan; 2 Department of Psychiatry and Behavioral Sciences, Johns Hopkins University School of Medicine, Baltimore, Maryland, United States of America; 3 Department of Regenerative Medicine and Stem Cell Biology, Faculty of Medicine, University of Tsukuba, Tsukuba, Ibaraki, Japan; 4 Department of Otorhinolaryngology Head and Neck Surgery, Faculty of Medical Sciences, University of Fukui, Fukui City, Fukui, Japan; 5 Department of Diagnostic Pathology, Faculty of Medicine, University of Tsukuba, Tsukuba, Ibaraki, Japan; 6 DNAVEC Research Inc, Tsukuba, Ibaraki, Japan; 7 Cell Engineering Division, RIKEN BioResource Center, Tsukuba, Ibaraki, Japan; 8 Japan Science and Technology Agency, Core Research for Evolutional Science and Technology (CREST), Chiyoda, Tokyo, Japan; University of Milan, Italy

## Abstract

The generation of induced pluripotent stem cells (iPSCs) by introducing reprogramming factors into somatic cells is a promising method for stem cell therapy in regenerative medicine. Therefore, it is desirable to develop a minimally invasive simple method to create iPSCs. In this study, we generated human nasal epithelial cells (HNECs)-derived iPSCs by gene transduction with Sendai virus (SeV) vectors. HNECs can be obtained from subjects in a noninvasive manner, without anesthesia or biopsy. In addition, SeV carries no risk of altering the host genome, which provides an additional level of safety during generation of human iPSCs. The multiplicity of SeV infection ranged from 3 to 4, and the reprogramming efficiency of HNECs was 0.08–0.10%. iPSCs derived from HNECs had global gene expression profiles and epigenetic states consistent with those of human embryonic stem cells. The ease with which HNECs can be obtained, together with their robust reprogramming characteristics, will provide opportunities to investigate disease pathogenesis and molecular mechanisms in vitro, using cells with particular genotypes.

## Introduction

Induced pluripotent stem cells (iPSCs) are generated from somatic cells by transducing them with reprogramming factors [1]. Initially, human dermal fibroblasts were used to derive human iPSCs (hiPSCs) [2], [3], and the majority of iPSC research in humans has focused on fibroblasts as a source of somatic cells. However, recent studies have shown that other human somatic cells can be used to generate iPSCs such as those from blood [4], [5], [6], [7], teeth [8], adipose tissues [9], and oral mucosa [10]. Obtaining these cells from the aforementioned sources, except blood, requires biopsy with local anesthesia, making it cumbersome for generating patient-specific stem cells. Additionally, although obtaining blood cells does not require local anesthesia, the rearrangement of the T-cell receptor (TCR) chain genes in T cells and the VDJ region in B cells means that they are not identical to naïve lymphocytes at the genomic level.

In the present study, we generated iPSC cells using human nasal epithelial cells (HNECs). This is a less invasive method to obtain human somatic cells, since neither anesthesia nor biopsy are required. In addition, we used Sendai virus (SeV) vectors to introduce reprogramming factors. SeV is an important respiratory pathogen of rats and mice, and it has been reported that SeV vectors efficiently transduce the respiratory tract of mice as well as humans [11]. Therefore, we speculated that HNECs would be highly amenable to efficient gene transduction with SeV vectors.

## Results

**Figure 1:**
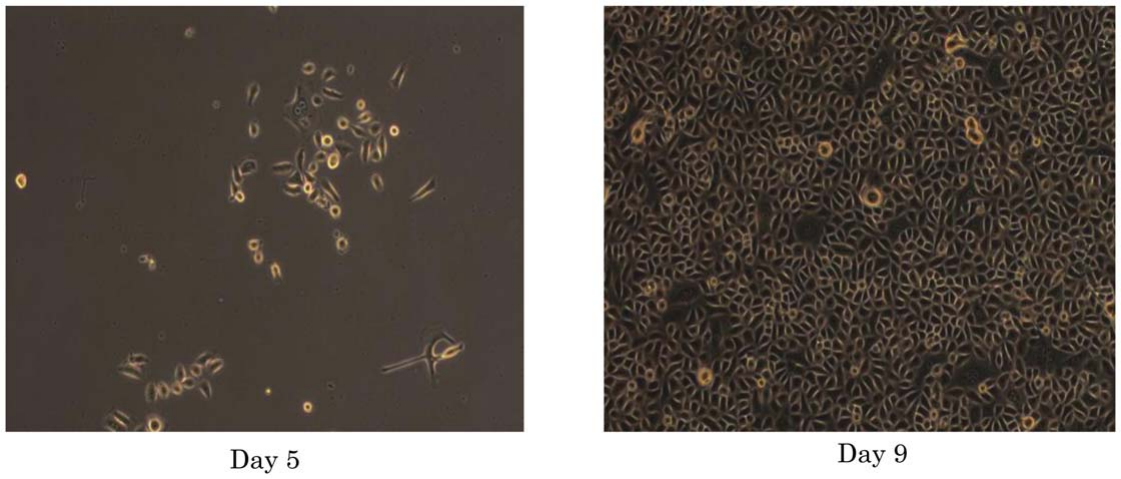
Primary culture of human nasal epithelial cells (HNEC). Bright-field images 5 daysaftercell sampling (left),and 9 days after sampling with early epithelioid morphology (right).

Freshly obtained HNECs were maintained on collagen-coated matrix, and they got attached within 4–6 hours, forming small colonies. The HNECs reached confluence within2 weeks (Figure 1) with typical epithelial morphology. We also confirmed that primary HNECs can be cultured and expanded after cryopreservation in liquid nitrogen.

**Figure 2:**
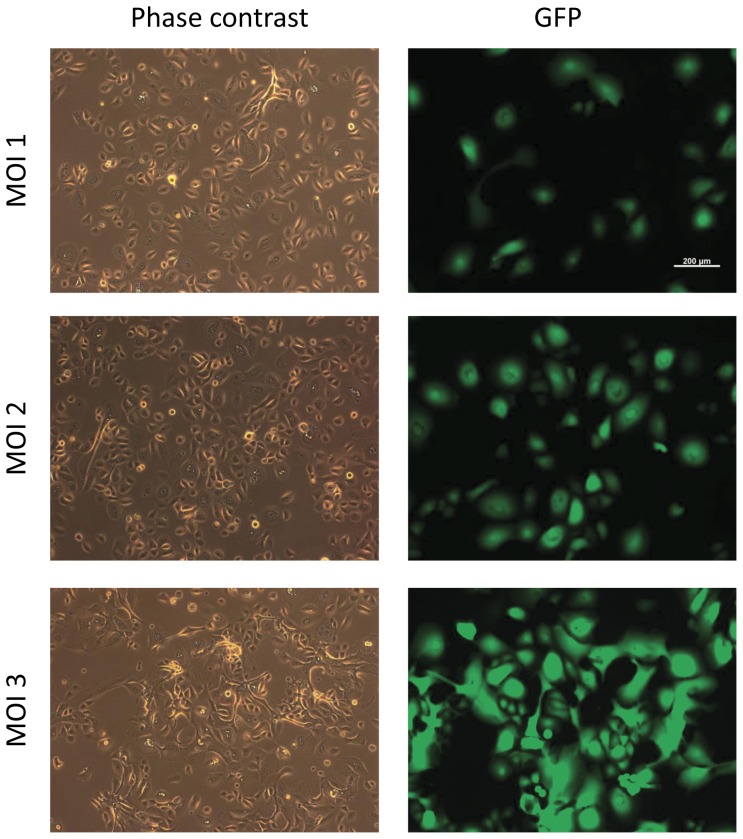
Expression of GFP in HNECs following various multiplicities of infection (MOI). Induction of GFP protein with MOI = 1 (top), MOI = 2, (middle), and MOI = 3 (bottom). GFP expression was observed when MOI = 1 and 2, but the expression was stronger when MOI = 3.

We first determined the infection efficiency using a SeV vector that expressed green fluorescent protein. HNECs seeded at 1.0×10^5^ cells per 35-mm dish were infected by green fluorescent protein vectors over a range of multiplicities of infection (MOI, number of viral particles per cell; Figure 2). We determined that a MOI of 3 or 4 was sufficient to induce the transgenes for HNECs.

**Figure 3:**
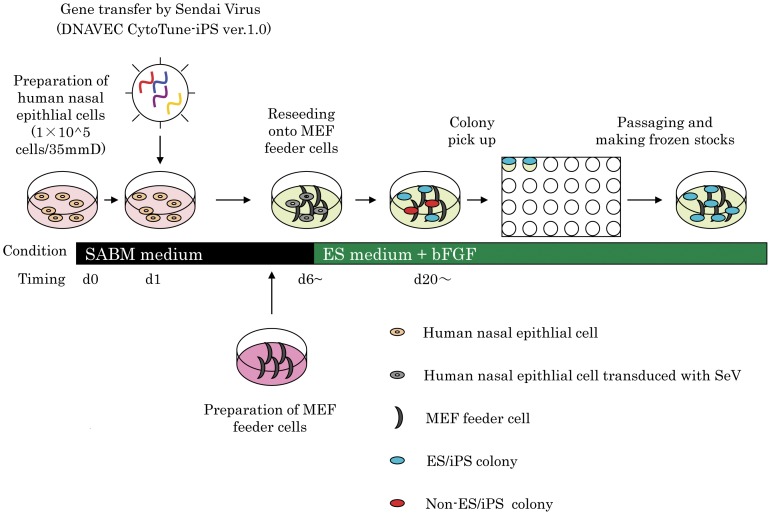
Schema of generation of iPS from HNECs.

**Figure 4:**
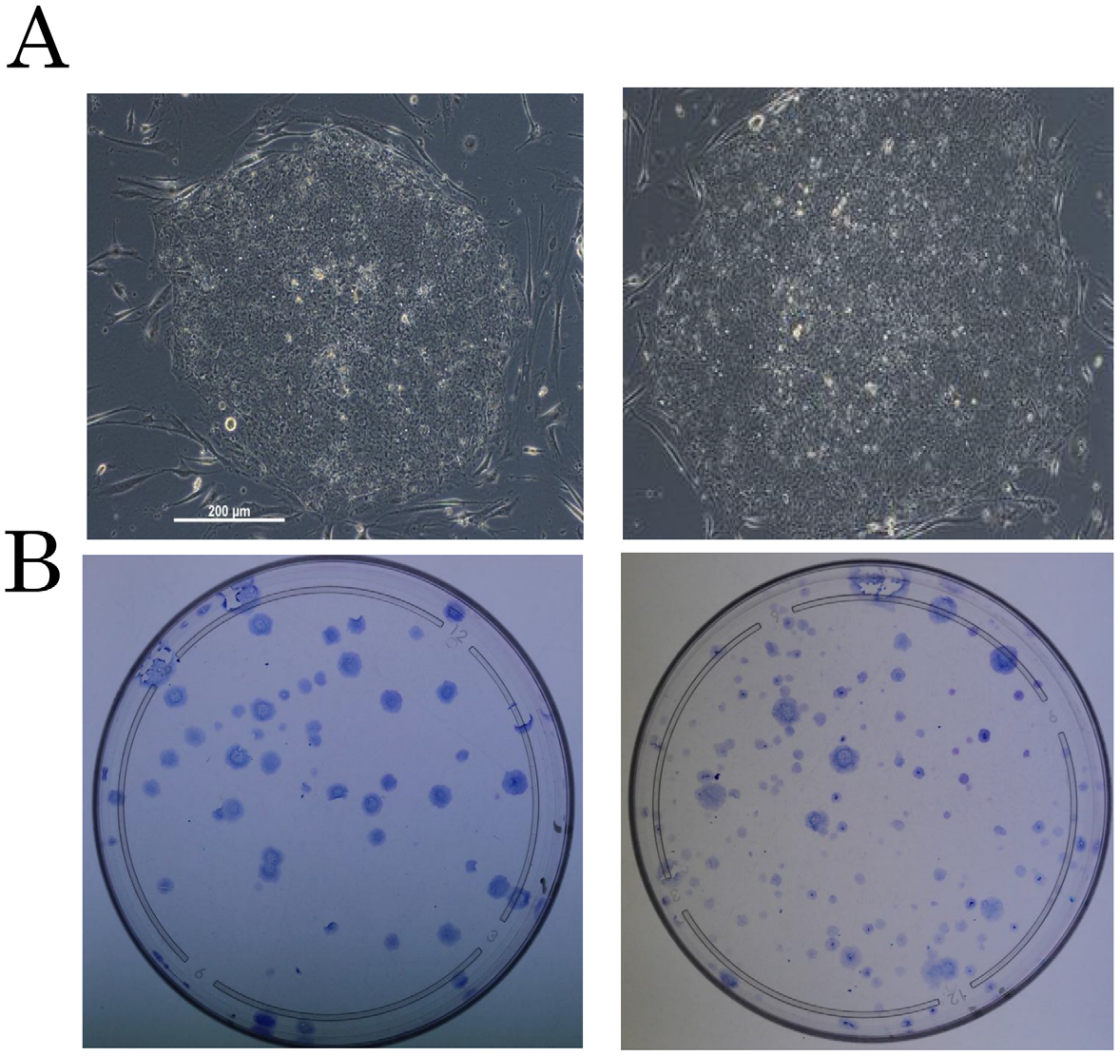
Morphologies of HNEC-derived iPS. A. Colonies generated from HNECs show a round shape with large nucleoli and scant cytoplasm, similar to the morphologyof human ES cells. B. Crystal violet staining of growing cells. Efficiency of iPS cell generation is 0.75% at MOI 3 (left), and 0.1% at MOI 4 (right).

The scheme for generation of iPS from HNECs is presented in Figure 3. We observed the appearance of colonies with an embryonic stem (ES) cell-like morphology at 20 days after infection of SeV vectors carrying 4 reprogramming factors (Figure 4A), and reprogramming efficiency was 0.1% at MOI 4, and 0.075% at MOI 3 (Figure 4B).

**Figure 5:**
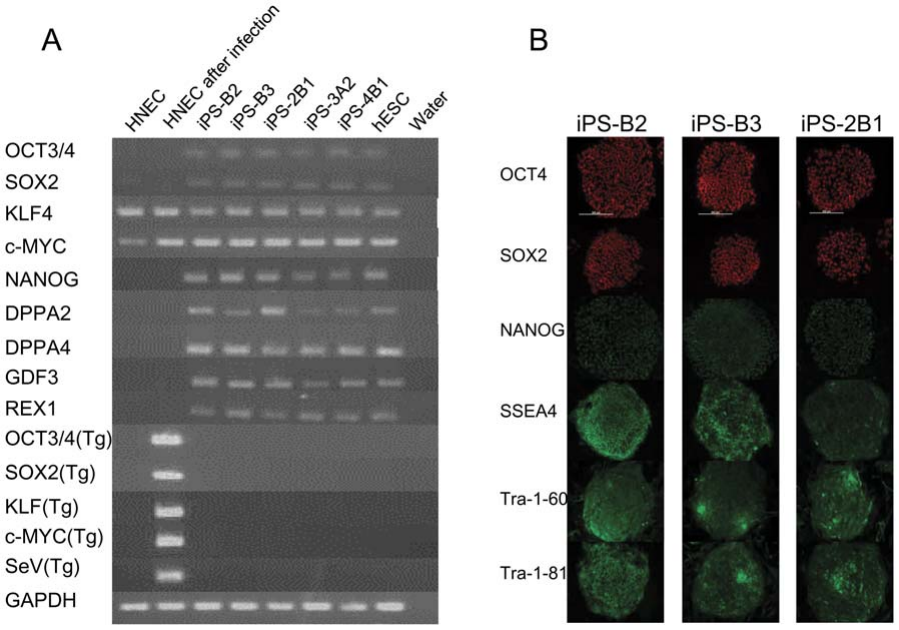
Gene/Protein expressions of HNEC-derived iPS. A. RT-PCR analyses for the hESC marker genes *NANOG*, *OCT3/4*, *SOX2*, *KLF4*, c*-MYC*, *GDF3*, *REX31*, *DPPA2*, and *DPPA4* and the transgenes *OCT3/4*, *SOX2*, *KLF4*, and *c-MYC*. B. Immunofluorescence staining for pluripotency and surface markers (OCT4, SOX2, NANOG,SSEA4,Tra-1-60, and Tra-1-81). Scale bars indicate 500 µm. Red: Alexa 568, Green: Alexa488.

**Figure 6:**
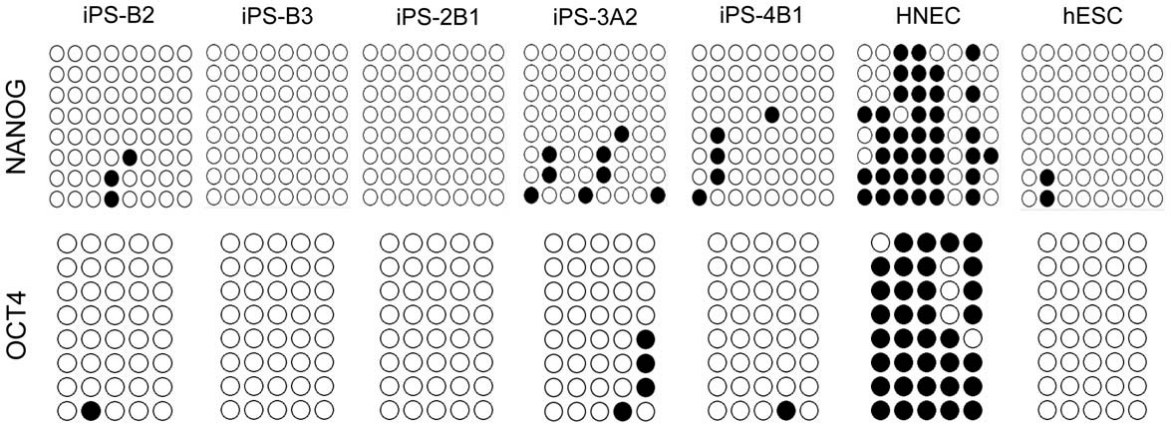
Methylation analysis of the promoter region. Bisulfite sequencing analysis of the *NANOG* and *OCT3/4* promoter regions in HNECs, hESCs, and HNEC-derived iPS cells B2, B3, 2B1, 3A2, and 4B1 are shown. Open circles indicate unmethylated CpGs, while closed circles indicate methylated CpGs.

**Figure 7:**
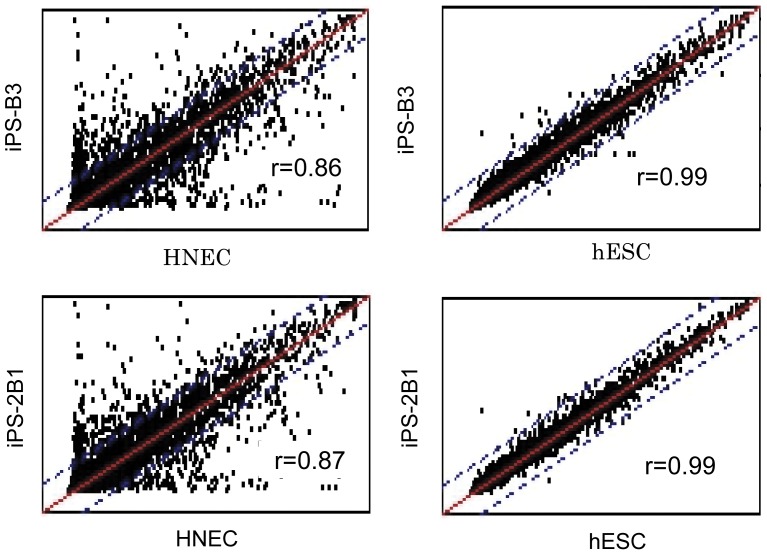
Global gene expression analysis. Gene expression patterns were compared between HNEC-derived iPS cells (iPS-B3) and HNECs (left, top), between HNEC-derived iPS cells (iPS-2B1) and HNECs (left, bottom), between HNEC-derived iPS cells (iPS-B3) and human ES cells (hESC, right, top), and between HNEC-derived iPS cells (iPS-2B1) and hESCs (right, bottom). Blue dotted lines indicate 2-fold changes. r represents the correlation coefficient.

To characterize colonies generated from SeV-infected HNECs, we picked a total of 74 colonies, and randomly chose 7 lines (iPS-B2, iPS-B3, iPS-C6, iPS-2B1, iPS-2B6, iPS-3A1, and iPS-4B1). We first performed a high-density single nucleotide polymorphism (SNP) genotyping assay to evaluate structural variations of HNEC-derived colonies. As shown in Figure S1, chromosomal duplications were observed on chromosome 2p in iPS-2B6 and chromosome 12p in iPS-C6. We excluded these colonies from further analysis. None of the other 5 colonies (iPS-B2, iPS-B3, iPS-2B1, iPS-3A1, and iPS-4B1) harbored duplications or deletions on chromosomes, and the genotype concordance rate in each colony with those of original HNECs was greater than 99.99%. This value was similar to the rate of technical replicates (i.e., concordance rate of the same genomic DNA), showing that colonies were derived from parental HNECs. Then, we examined the gene expression of the reprogramming factors and the expression of SeV vectors in HNEC-derived colonies (Figure 5). We used a temperature-sensitive mutant SeV vector in these experiments in order to shut off transgenes efficiently by temperature shift [12]. Colonies generated from HNECs showed endogenous *OCT4*, *SOX2*, *KLF4*, and *c-MYC* gene expression levels that were similar to those of human ES cells, and we confirmed that SeV-derived gene expressions were not detected by reverse transcription-polymerase chain reaction (RT-PCR) using SeV-specific primers (Figure 5A). Protein expression of pluripotency markers was confirmed by immunofluorescence staining (Figure 5B and Figure S2). DNA methylation analysis revealed that CpG dinucleotides at the *OCT4* and *NANOG* promoter region in the HNEC-derived colonies were demethylated while those in original HNECs were mostly methylated (Figure 6). Furthermore, global gene expression pattern in the HNEC-derived cell lines strongly correlated with that of human ES cells (r = 0.99, Figure 7), while correlation of gene expression between HNEC-derived cell lines and parental HNECs was weak (r = 0.87 in iPS-B3 and r = 0.86 in iPS-2B1).

In order to evaluate the differentiation potential of HNEC-derived iPSCs, we performed in vitro differentiation using HNEC-derived cell lines. We generated embryoid bodies (EBs), which were spontaneously differentiated for 10∼13 days. RT-PCR analysis revealed that these cells were positive for three germ cell markers (Figure S3), although the gene expression pattern of these cells varied in different HNEC-derived iPS cell lines. Next, in order to investigate in *vivo* differentiation, we injected HNEC-derived cell lines into immunocompromised mice and evaluated their ability to form teratomas. In the experiments, we used 4 cell lines (iPS-B2, iPS-B3, iPS-2B1, and iPS-3A2). Histological examination of the teratomas revealed that tissues were from the endoderm, mesoderm, and ectoderm lineages, and we observed neural and epithelial tissues, muscle, cartilage, bone, gut-like structures, and various glandular structures (Figure S4).

## Discussion

In the present study, we demonstrated that efficient reprogramming of HNECs can be achieved without integration of the reprogramming transgenes or a viral genome. iPS cells generated in the present study showed characteristics similar to human ES cells, and had the potential to differentiate into the 3 germinal layers of endoderm, mesoderm, and ectoderm in a teratoma formation assay *in vivo*.

One of the many benefits that iPS technology offers is the development of robust and personalized models of human disease. iPSCs can be generated from subjects with various genetic diseases [13], [14]. Skin fibroblasts are frequently used to generate iPSCs. However, because this requires skin biopsy with local anesthesia, it can be challenging to recruit donors. An alternative is to use blood cells as a source for iPSCs. This has been performed using both peripheral B cells [15], and T cells [5], [7], but in this scenario, the genomic region of TCR chain genes in T cells and the VDJ region in B cells are rearranged. Therefore, iPSCs derived from T and B cells can exhibit only 1 pattern of the TCR gene and VDR genomic regions, respectively. CD34-positive cells have also been used to generate iPSCs [6], [16], [17]. This has a potential advantage over using the mature T and B cells, since CD34-positive cells do not have their genomes rearranged. However, these cells comprise only a small proportion (0.1%) of total peripheral hematopoietic cells, which poses a challenge for the separation of pure CD34-positive cells. Our current method using HNECs maintains the genome in the same state as the original somatic cells, does not require complicated methods for culture or a high MOI for gene transduction, and achieves reprogramming efficiencies similar to those found using skin fibroblasts (0.08–0.13% [12]). Because of the minimal invasiveness involved in obtaining these somatic cells and their amenability to reprogramming, using SeV vectors to reprogram HNECs may be a more suitable approach for generating patient-specific iPS cells. This will provide opportunities to investigate disease pathogenesis and molecular mechanisms in *vitro*, using cells with particular genotypes.

## Materials and Methods

### Ethics statement

This study was approved by the Ethical Committee of University of Tsukuba and followed International guidelines (i.e., the Helsinki Declaration). Informed written consent was taken from a tissue sample donor. All animal work was conducted according to university and international guidelines.

### Primary cell culture

HNECs were obtained from a healthy female donor by the brushing technique. Nasal brushing was performed by an otolaryngologist using a soft sterile brush (Medical Packaging Camarillo, CA). A brushing was performed in both nostrils by a gentle circular movement. Brushes were immersed in 3 mL of small airway epithelial cells basal medium (SABM) (Lonza, Basel, Switzerland) with small airway epithelial cell growth medium (SAGM) SingleQuots (Lonza, Basel, Switzerland) and 100 U penicillin and 0.1 mg/mL streptomycin (Sigma-Aldrich, St. Louis, MO), followed by a manual shake and pipetting. SABM containing HNECs were seeded on type Ι collagen-coated 35-mm dishes (BD Biocoat, BD, Franklin Lakes, NJ), and cultured at 37°C in an atmosphere of 5% CO_2_. After 24 and 48 h of culture, non-adherent HNECs were collected and transferred to a new type Ι collagen-coated dish. SABM was changed every 2 days, and cells were cultured with SABM with SAGM SingleQuots and Reagent Pack (Lonza, Basel, Switzerland).

### Induction of pluripotent stem cells

After 2 weeks of culture, HNECs were collected and 1.0×10^5^ cells were transferred to a new 35-mm dish coated with type Ι collagen (BDBiocoat, BD, Franklin Lakes, NJ), and cultured at 37°C in an atmosphere of 5% CO_2_ overnight. HNECs were infected with SeV vectors (DNAVEC, Tsukuba, Japan) that individually carried each of *OCT3/4*, *SOX2*, *KLF4*, and *c-MYC* at an MOI of 3 or 4. Infected cells were cultured in SABM supplemented with SAGM SingleQuots. After 24 h of infection, the medium was changed to fresh medium. At 6–7 days after infection, 1.0–2.0×10^5^ cells were collected and transferred to a 10 cm-dish containing mitomycin C-inactivated mouse embryonic fibroblast (MEF) feeder cells (1.5×10^6^ cells per dish). After an additional 24 h of incubation, the medium was changed to hiPSC medium, which consisted of Dulbecco's modified Eagle medium/F12 medium (Life Technologies, Carlsbad, CA) supplemented with 20% Knock-out Serum Replacement (KSR; Life Technologies, Carlsbad, CA), 2 mMl-glutamine (Life Technologies), 1 mM non-essential amino acids (Sigma-Aldrich, St. Louis, MO), 0.1 mM β-mercaptoethanol (Sigma-Aldrich, St. Louis, MO), and 4 ng/mL basic fibroblast growth factor (WAKO, Osaka, Japan). The hiPSC medium was changed every day. The generated iPSCs were maintained on mitomycin C-inactivated MEF feeder cells in hiPSC 3 medium, and the cells were passaged with CTK solution consisted of 0.25% trypsin, 1 mg/mL collagenase IV, 20% KSR (all from Life Technologies, Carlsbad, CA), 20 mM CaCl_2_ (Wako) in PBS(-) (Life Technologies, Carlsbad, CA).

### Reprogramming efficiency

Reprogramming efficiency was calculated as the number of iPS colonies formed per number of infected cells seeded. iPS colonies were identified based on ES cell-like morphology and crystal violet staining. Crystal violet staining was performed with 0.1% crystal violet (Nakaraitesk, Kyoto, Japan) in methanol, as described previously [18].

### SNP genotyping

Genomic DNA was isolated from HNEC-derived iPS cells by DNeasy Blood & Tissue kit (Qiagen, Hilden, Germany). High-density SNP genotyping was performed using Human610-Quad (Illumina, San Diego, CA) that contains more than 610,000 rationally selected tag SNPs and markers per sample, according to the manufacturer's protocol. The scanned image was imported into GenomeStudio (Illumina) for analysis. Genotype calls and analysis were performed by GenomeStudio (Illumina).

### Immunofluorescence staining

Immunofluorescence staining was performed using the following primary antibodies:anti-NANOG (Cell Signaling Technology, Danvers, MA), StemLite™Pluripotency kit (anti-OCT4, anti-Sox2, anti-SSEA4, anti-TRA-1-60, anti-TRA-1-81; Cell Signaling Technology). The secondary antibodies used were: anti-rabbit IgG, anti-mouse IgG and IgM conjugated with AlexaFluor 488 or AlexaFluor 568 (Life Technologies, Carlsbad, CA) and anti-rabbit IgG(H+L), fragments conjugated with AlexaFluor 488(Cell Signaling Technology). Cell nuclei were stained with DAPI (Vector Laboratories).The fluorescence signals were detected using a conventional fluorescence laser microscope (ZEISS-HBO-100; Carl Zeiss AG, Oberkochen, Germany).

### RT-PCR

Total RNA was isolated using the RNeasy Mini kit (Qiagen), according to the manufacturer's instructions. The concentration and purity of the RNA were determined using the ND-1000 spectrophotometer (Nanodrop, Wilmington, DE). The cDNA was synthesized using the T7 Oligo (dT) Primer (Ambion, Austin, TX), 1000 U/µL ReverTra Ace (TOYOBO, Osaka, Japan), 5000 U RNaseout™ recombinant ribonuclease inhibitor (Life Technologies). RT-PCR was performed with AmpliTaq Gold 360 Master Mix (Life Technologies). The primers used for RT-PCR are listed in Table S1.

### Global gene expression analysis

Total RNA was isolated from HNEC-derived iPS cells using the RNeasy Mini Kit. We used Illumina Bead Array with single-color array (Illumina) as a microarray platform. For the Illumina BeadArray assay, cRNA was synthesized with an Illumina RNA Amplification kit (Life Technologies), according to the manufacturer's instructions. In brief, 500 ng of total RNA were reverse transcribed to synthesize first- and second-strand cDNA, purified with spin columns, and then *in vitro* transcribed to synthesize biotin-labeled cRNA. A total of 750 ng biotin-labeled cRNA was hybridized to each Illumina Human-Ref8 v3.0 BeadChip array (Illumina) at 55°C for 18 h. The hybridized BeadChip was washed and labeled with streptavidin-Cy3 (GE Healthcare, Buckinghamshire, UK) and then scanned with the Illumina BeadStation 500 System (Illumina). The scanned image was imported into GenomeStudio software (Illumina) for analysis. Twenty-two thousand transcripts representing 8 whole-genome samples can be analyzed on a single BeadChip. GenomeStudio output of the microarray data was processed with lumi package for the R language [19] on R version 2.10.0 (http://www.R-project.org/).

### Bisulfite sequencing

Genomic DNA was isolated from HNECs, human ES cells, and iPS cells derived from HNECs using DNeasy Blood & Tissue kit (Qiagen), and was treated with sodium bisulfite using EpiTect Bisulfite kit (Qiagen), according to the manufacturer's instructions. Converted DNA was used as the template for PCR using primer sets previously described to amplify the promoter regions of *OCT4* [20] and *NANOG* [2]. The purified PCR products were TA-cloned into pCR4-TOPO vector using TOPO TA Cloning Kit for Sequencing (Life Technologies). The insert sequences of randomly picked clones were analyzed using the ABI 3130 DNA analyzer (Life Technologies).

### In vitro differentiation

iPS cells were cultured on Petri dishes for one days in hiPSC medium, then were cultured free floating to induce EBs for 10–13 days in EB medium, consisting of DMEM/Ham's F12 containing5% KSR, 2 mMl-glutamin, 1×10^−4^ M nonessential amino acids, and 1×10^−4^ M 2-mercaptoethanol.

### Teratoma formation

All mouse procedures were conducted in compliance with institutional animal use guidelines. hiPSCs grown on MEF feeder layers were collected by CTK solution into tubes, and centrifuged, and the pellets were suspended in 10 µM Y-27632 (Wako) in cold Hanks' Balanced Salt Solution (Life Technologies). The cells from a confluent 60-mm dish were injected (5×10^6^ cells in 200 µL per injection) into 1 testis of SCID mice (Japan Charles River, Yokohama, Japan) using a 1-mL syringe. At 8–10 weeks post injection, teratomas were dissected, fixed in 4% paraformaldehyde, and embedded in paraffin. The sections were stained with hematoxylin and eosin.

## Supporting Information

Figure S1 **Genome-wide SNP genotyping analysis.** A. Log R ratio and B allele frequency plots of chromosome 12 in iPS-C6. B. Log R ratio and B allele frequency of chromosome 2 in iPS-2B6. Red circles indicate duplicated chromosomal regions.
(TIF)

Figure S2 **Protein expressions of HNEC-derived iPS.** Immunofluorescence staining for pluripotency and surface markers (OCT4, SOX2, NANOG,SSEA4,Tra-1-60, and Tra-1-81). All cells were also stained with DAPI and images were merged with lineage markers.Scale bars indicate 100 µm. Yellow: Alexa 546, Green: Alexa488, Blue: Alexa460.
(TIF)

Figure S3 **RT-PCR analysis of EBs generated from HNEC-derived iPS cells.** RT-PCR results for ectoderm (paired box 6 (PAX6) and microtubule-associated protein 2 (MAP2)), mesoderm (BRACHYURY/T and homeobox 1 (MSX1)), and endoderm (SRY-box 17 (SOX17) and alpha-fetoprotein(AFP)) are presented. 1,2,3, and 4 correspond to iPS-B2, iPS-B3, iPS-2B1, and the negative control, respectively.
(TIF)

Figure S4 **Differentiation of 3 embryonic germ layers from HNEC-derived iPS cells.** Endoderm (left), mesoderm (middle), and ectoderm (right) generated from iPS-B2, iPS-B3, and iPS-2B1 and iPS3A2, respectively.
(TIF)

Table S1 **Primers used for RT-PCR.**
(XLS)

## References

[1] Zaehres H, Scholer HR (2007) Induction of pluripotency: from mouse to human. Cell 131: 834–835. 18045526 10.1016/j.cell.2007.11.020

[2] Takahashi K, Tanabe K, Ohnuki M, Narita M, Ichisaka T, et al (2007) Induction of pluripotent stem cells from adult human fibroblasts by defined factors. Cell 131: 861–872. 18035408 10.1016/j.cell.2007.11.019

[3] Yu J, Vodyanik MA, Smuga-Otto K, Antosiewicz-Bourget J, Frane JL, et al (2007) Induced pluripotent stem cell lines derived from human somatic cells. Science 318: 1917–1920. 18029452 10.1126/science.1151526

[4] Chou BK, Mali P, Huang X, Ye Z, Dowey SN, et al (2011) Efficient human iPS cell derivation by a non-integrating plasmid from blood cells with unique epigenetic and gene expression signatures. Cell Res 21: 518–529. 21243013 10.1038/cr.2011.12 PMC3193421

[5] Loh YH, Hartung O, Li H, Guo C, Sahalie JM, et al (2010) Reprogramming of T cells from human peripheral blood. Cell Stem Cell 7: 15–19. 20621044 10.1016/j.stem.2010.06.004 PMC2913590

[6] Mack AA, Kroboth S, Rajesh D, Wang WB (2011) Generation of induced pluripotent stem cells from CD34+ cells across blood drawn from multiple donors with non-integrating episomal vectors. PLoS One 6: e27956. 22132178 10.1371/journal.pone.0027956 PMC3222670

[7] Seki T, Yuasa S, Oda M, Egashira T, Yae K, et al (2010) Generation of Induced Pluripotent Stem Cells from Human Terminally Differentiated Circulating T Cells. Cell Stem Cell 7: 11–14. 20621043 10.1016/j.stem.2010.06.003

[8] Tamaoki N, Takahashi K, Tanaka T, Ichisaka T, Aoki H, et al (2010) Dental pulp cells for induced pluripotent stem cell banking. J Dent Res 89: 773–778. 20554890 10.1177/0022034510366846

[9] Sun N, Panetta NJ, Gupta DM, Wilson KD, Lee A, et al (2009) Feeder-free derivation of induced pluripotent stem cells from adult human adipose stem cells. Proc Natl Acad Sci U S A 106: 15720–15725. 19805220 10.1073/pnas.0908450106 PMC2739869

[10] Miyoshi K, Tsuji D, Kudoh K, Satomura K, Muto T, et al (2010) Generation of human induced pluripotent stem cells from oral mucosa. J Biosci Bioeng 110: 345–350. 20547351 10.1016/j.jbiosc.2010.03.004

[11] Yonemitsu Y, Kitson C, Ferrari S, Farley R, Griesenbach U, et al (2000) Efficient gene transfer to airway epithelium using recombinant Sendai virus. Nat Biotechnol 18: 970–973. 10973218 10.1038/79463

[12] Ban H, Nishishita N, Fusaki N, Tabata T, Saeki K, et al (2011) Efficient generation of transgene-free human induced pluripotent stem cells (iPSCs) by temperature-sensitive Sendai virus vectors. Proc Natl Acad Sci U S A 108: 14234–14239. 21821793 10.1073/pnas.1103509108 PMC3161531

[13] Park IH, Arora N, Huo H, Maherali N, Ahfeldt T, et al (2008) Disease-specific induced pluripotent stem cells. Cell 134: 877–886. 18691744 10.1016/j.cell.2008.07.041 PMC2633781

[14] Yusa K, Rashid ST, Strick-Marchand H, Varela I, Liu PQ, et al (2011) Targeted gene correction of alpha1-antitrypsin deficiency in induced pluripotent stem cells. Nature 478: 391–394. 21993621 10.1038/nature10424 PMC3198846

[15] Hanna J, Markoulaki S, Schorderet P, Carey BW, Beard C, et al (2008) Direct reprogramming of terminally differentiated mature B lymphocytes to pluripotency. Cell 133: 250–264. 18423197 10.1016/j.cell.2008.03.028 PMC2615249

[16] Liu T, Zou G, Gao Y, Zhao X, Wang H, et al (2012) High efficiency of reprogramming CD34+ cells derived from human amniotic fluid into induced pluripotent stem cells with Oct4. Stem Cells Dev 10.1089/scd.2011.0715 22264161

[17] Takenaka C, Nishishita N, Takada N, Jakt LM, Kawamata S (2010) Effective generation of iPS cells from CD34+ cord blood cells by inhibition of p53. Exp Hematol 38: 154–162. 19922768 10.1016/j.exphem.2009.11.003

[18] Takahashi K, Okita K, Nakagawa M, Yamanaka S (2007) Induction of pluripotent stem cells from fibroblast cultures. Nat Protoc 2: 3081–3089. 18079707 10.1038/nprot.2007.418

[19] Du P, Kibbe WA, Lin SM (2008) lumi: a pipeline for processing Illumina microarray. Bioinformatics 24: 1547–1548. 18467348 10.1093/bioinformatics/btn224

[20] Freberg CT, Dahl JA, Timoskainen S, Collas P (2007) Epigenetic reprogramming of OCT4 and NANOG regulatory regions by embryonal carcinoma cell extract. Mol Biol Cell 18: 1543–1553. 17314394 10.1091/mbc.E07-01-0029 PMC1855029

