# Supplementary figures and images for: Generation of Induced Pluripotent Stem Cells from Human Nasal Epithelial Cells Using a Sendai Virus Vector
Source: PLoS One. 2012 Aug 13;7(8):e42855. doi: 10.1371/journal.pone.0042855 (PMC3418281; doi:10.1371/journal.pone.0042855)

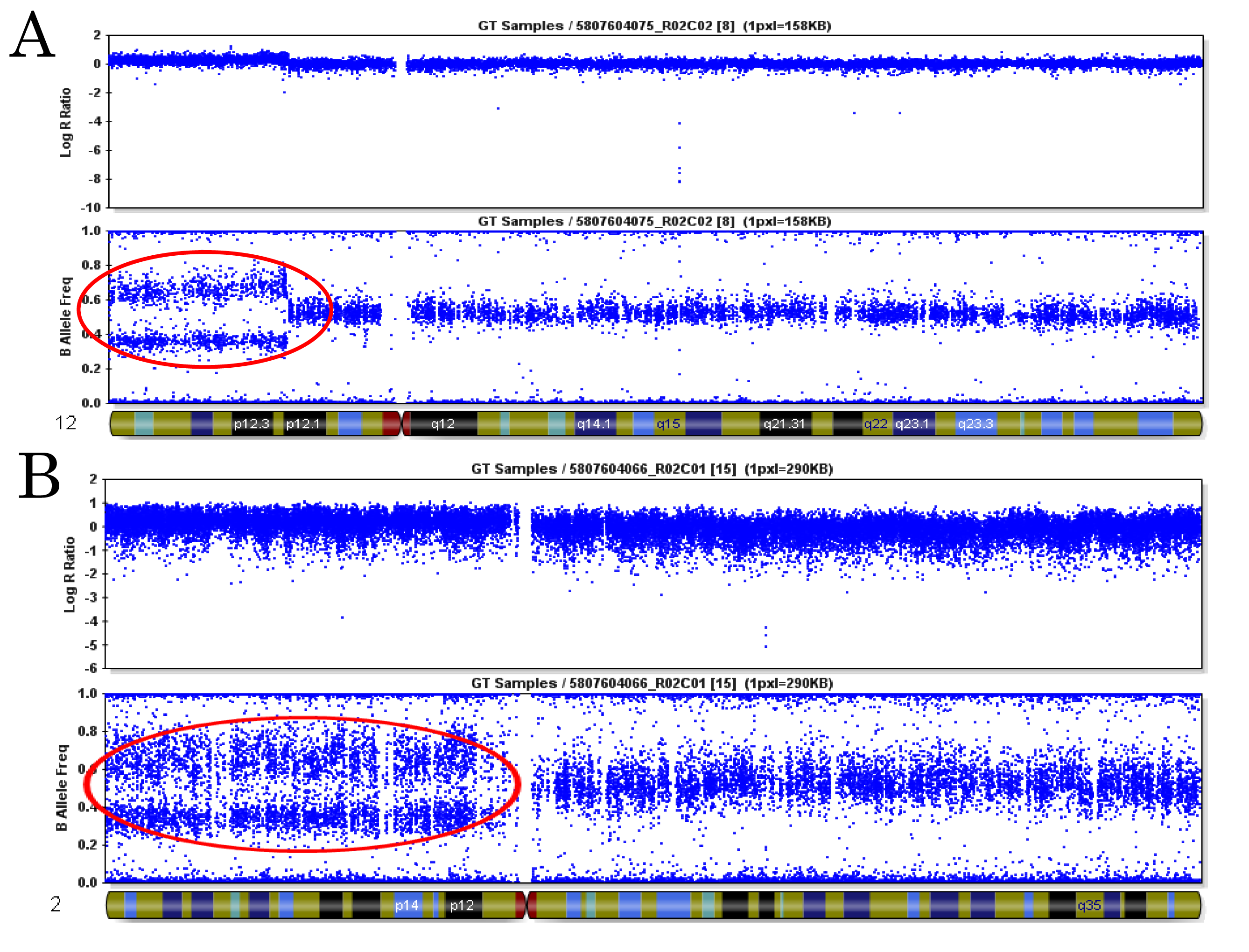

Supplement: Figure S1 — Genome-wide SNP genotyping analysis. A. Log R ratio and B allele frequency plots of chromosome 12 in iPS-C6. B. Log R ratio and B allele frequency of chromosome 2 in iPS-2B6. Red circles indicate duplicated chromosomal regions. (TIF) [file pone.0042855.s001.tif]

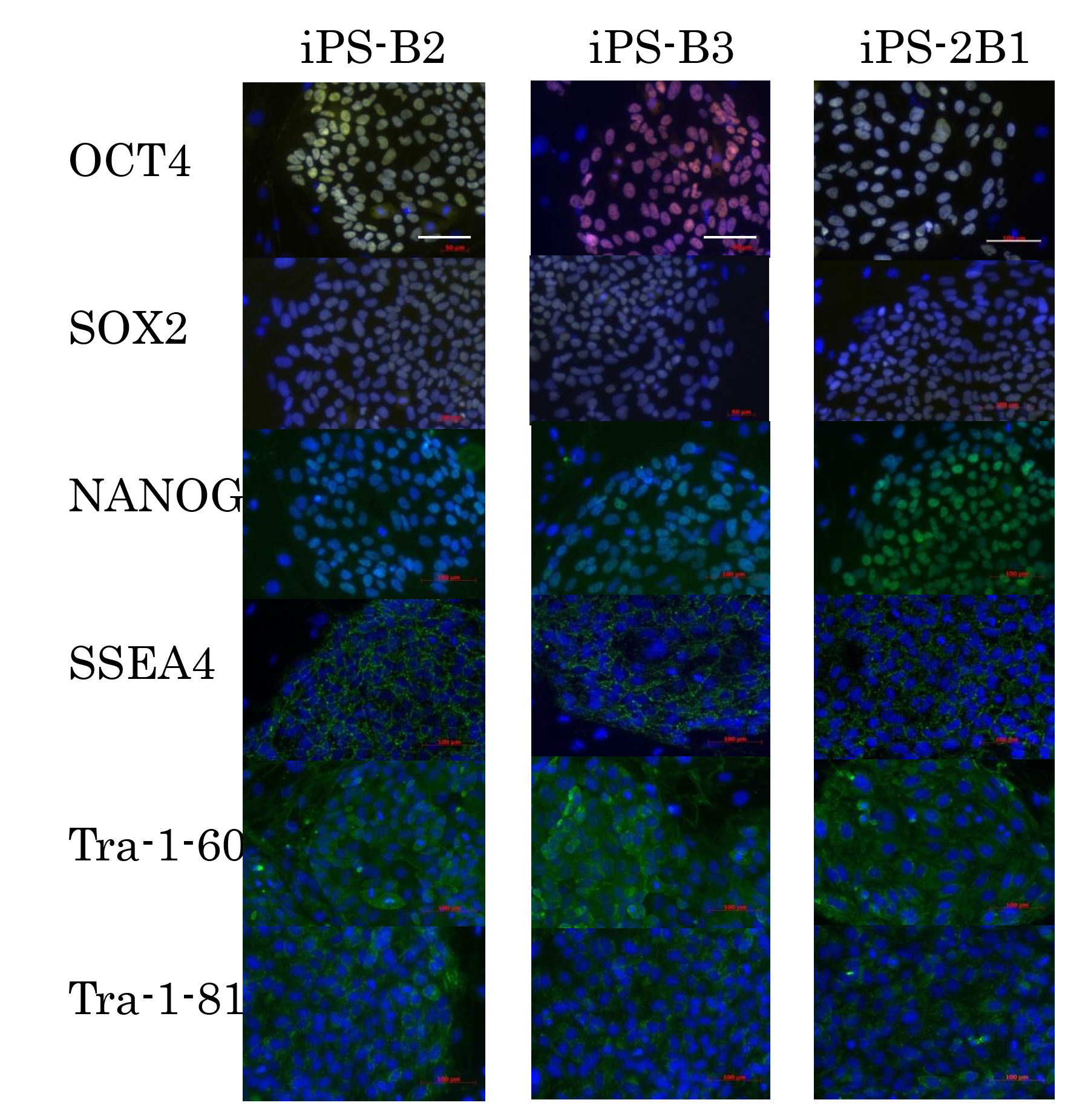

Supplement: Figure S2 — Protein expressions of HNEC-derived iPS. Immunofluorescence staining for pluripotency and surface markers (OCT4, SOX2, NANOG,SSEA4,Tra-1-60, and Tra-1-81). All cells were also stained with DAPI and images were merged with lineage markers.Scale bars indicate 100 µm. Yellow: Alexa 546, Green: Alexa488, Blue: Alexa460. (TIF) [file pone.0042855.s002.tif]

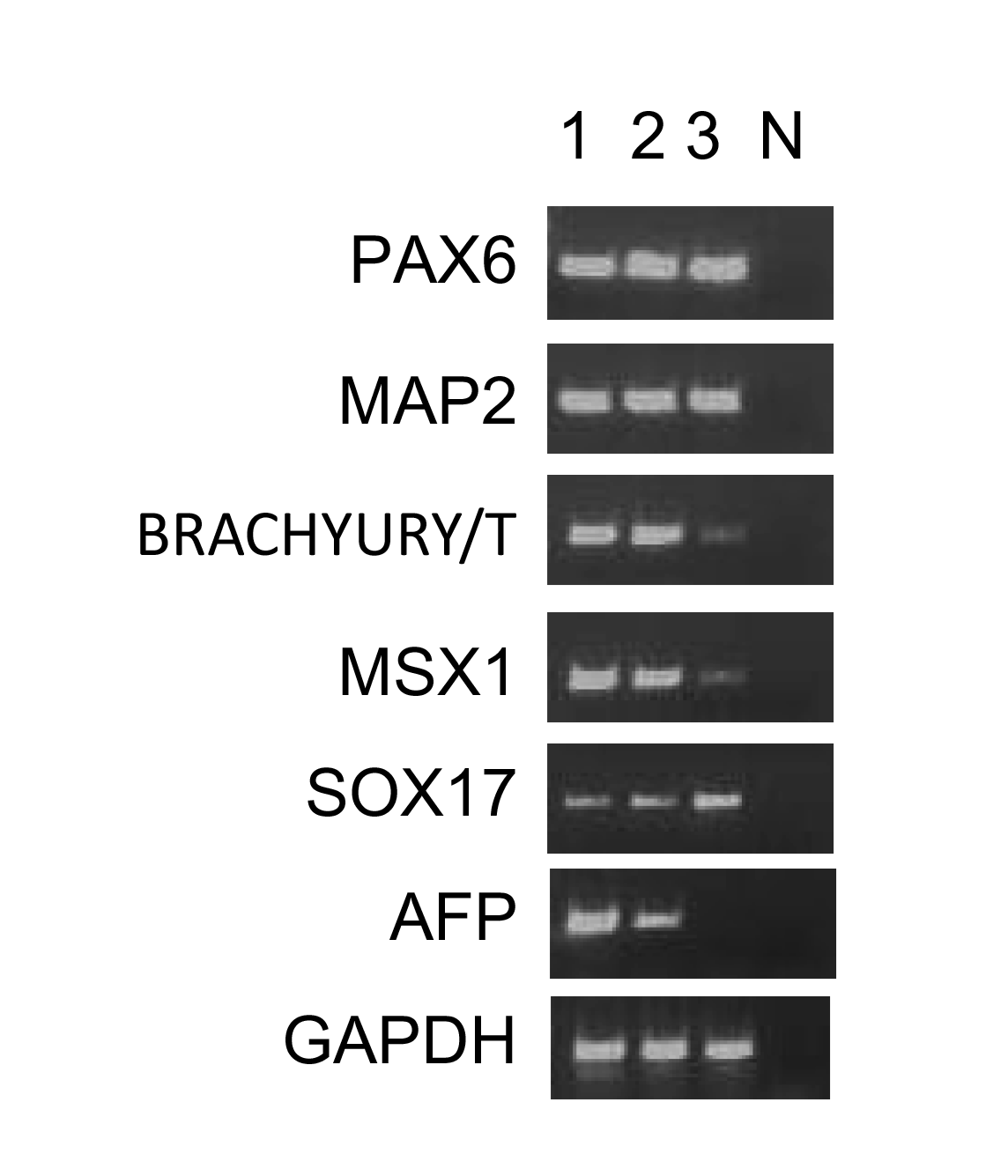

Supplement: Figure S3 — RT-PCR analysis of EBs generated from HNEC-derived iPS cells. RT-PCR results for ectoderm (paired box 6 (PAX6) and microtubule-associated protein 2 (MAP2)), mesoderm (BRACHYURY/T and homeobox 1 (MSX1)), and endoderm (SRY-box 17 (SOX17) and alpha-fetoprotein(AFP)) are presented. 1,2,3, and 4 correspond to iPS-B2, iPS-B3, iPS-2B1, and the negative control, respectively. (TIF) [file pone.0042855.s003.tif]

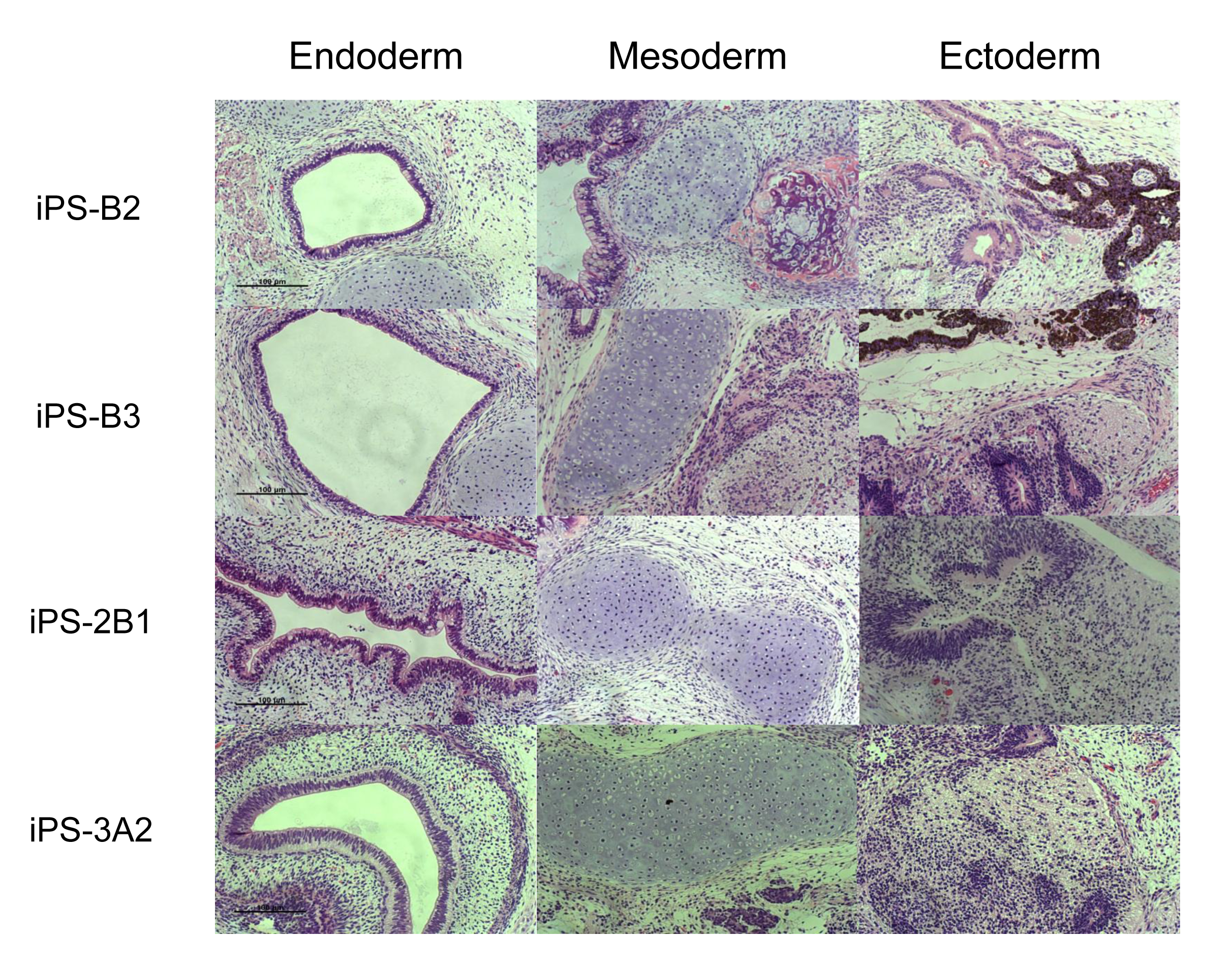

Supplement: Figure S4 — Differentiation of 3 embryonic germ layers from HNEC-derived iPS cells. Endoderm (left), mesoderm (middle), and ectoderm (right) generated from iPS-B2, iPS-B3, and iPS-2B1 and iPS3A2, respectively. (TIF) [file pone.0042855.s004.tif]
